# Reconstruction of a Large Defect at the Junction of the Medial Canthus and the Superior Nasal Sidewall

**DOI:** 10.7759/cureus.83188

**Published:** 2025-04-29

**Authors:** Samuel Stahly, Charles Dunn, Yasser Faraj, Alexander Dane

**Affiliations:** 1 Dermatology, Kansas City University Graduate Medical Education (KCU-GME) Consortium and Advanced Dermatology and Cosmetic Surgery (ADCS) Orlando, Orlando, USA

**Keywords:** advancement flap, face anatomy, general dermatology, mohs surgery, plastic surgery

## Abstract

A 66-year-old female active smoker with a history of nonmelanoma skin cancer underwent Mohs micrographic surgery for a basal cell carcinoma located at the junction of the superior nasal sidewall and medial canthus. The cancer was completely excised in two stages of Mohs surgery; however, the resulting surgical defect, measuring 1.5 x 1.6 cm, posed a challenging reconstruction. Several factors contributed to the complexity of the repair, including the lesion's large size, involvement of multiple cosmetic subunits, proximity to the free margin of the eye, and the patient’s smoking status. Common Mohs surgery repair techniques include direct approximation, secondary intention healing, advancement or rotational flaps, and full-thickness skin grafts. Each case requires an individualized approach, considering its unique characteristics. In this instance, a novel variation of an advancement flap was developed. An advancement flap is a surgical technique that moves adjacent tissue over a defect linearly. The island pedicle, a type of advancement flap, remains attached to its underlying blood supply to enhance survival. This technique relies on subcutaneous fat, limiting its use. A myocutaneous V-to-Y flap is a variant utilizing the vascular supply of an underlying muscle, making it suitable for areas with minimal subcutaneous tissue. The nasalis sling, commonly used for distal nose defects, exemplifies this approach. We report a novel myocutaneous V-to-Y flap variant using the glabellar musculature to repair a defect in the superior nasal sidewall and medial canthus. This technique demonstrates reliability in active smokers, leveraging the vascular supply of underlying muscles for improved survival. Additionally, it offers a superior tissue and texture match, as it uses adjacent tissue. This report aims to assist surgeons facing similar reconstructive challenges, as this technique yielded an excellent cosmetic outcome in our patient.

## Introduction

Surgical defects resulting from Mohs micrographic surgery can present significant reconstructive challenges, particularly in cosmetically sensitive areas such as the face. Patient characteristics and tumor location often complicate repair planning. Factors such as defect size and depth, proximity to free margins, patient age, and smoking status must be carefully evaluated [1]. Additionally, sex-based differences in nasal and facial dimensions should be considered to minimize complications [2]. Each case demands an individualized approach, tailored to its unique attributes.

Our case involved repairing a defect at the junction of the superior nasal sidewall and medial canthus near the glabellar complex. The proximity to a free margin and the patient’s active smoking status influenced the chosen repair technique. Local flaps frequently used in the glabellar area, such as transposition and rotational flaps, generally heal well but may cause pincushioning or result in scar lines that do not align with the natural glabellar rhytides [3]. Other options, such as full-thickness skin grafts and secondary intention healing, pose additional challenges. Skin grafts often yield suboptimal tissue matches, while smoking status may impair wound healing for both options [1,4,5].

Given these considerations, we opted for a variant of the island pedicle flap. Island pedicle flaps rely on an attached subcutaneous stalk for blood supply, enhancing tissue viability [6]. These flaps are advantageous due to their ability to use adjacent tissue with matching texture and color. However, their success depends on robust subcutaneous tissue [7]. The nasalis sling flap, a well-documented variant, utilizes the nasalis muscle’s vascular supply for distal nasal defect repairs [8]. By adapting these principles, we developed a novel myocutaneous V-to-Y advancement flap using the glabellar musculature to address the defect at the superior nasal sidewall and medial canthus.

This case report complies with the ethical standards set forth in the Declaration of Helsinki. Informed consent was obtained from the patient on July 23, 2024, for both the surgical procedure and the publication of this case report, including the use of anonymized medical data and any accompanying images. The patient’s identity has been protected to ensure confidentiality.

## Case presentation

**Figure 1:**
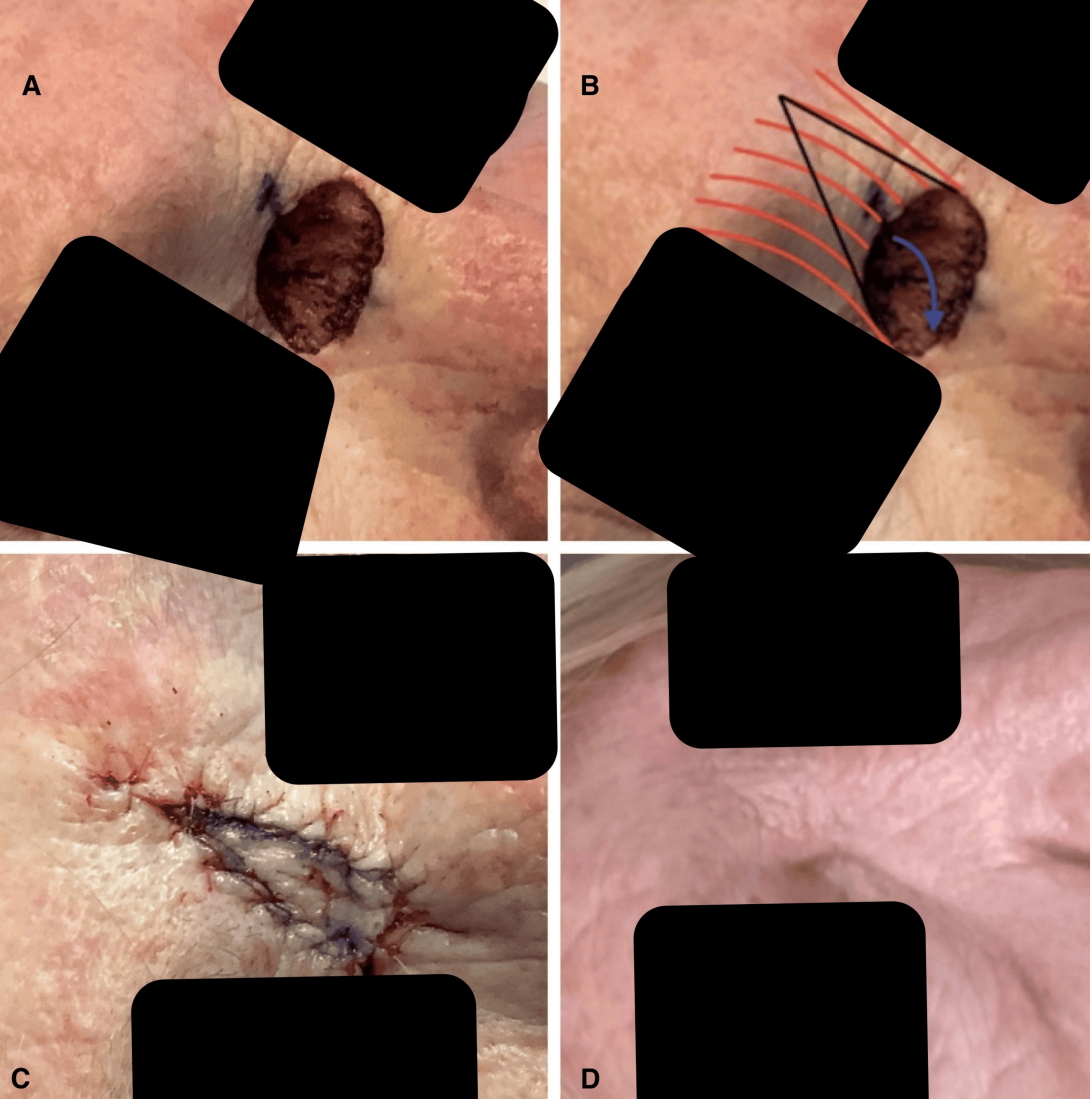
Four photos showing the various stages of the surgical repair. A: depicts the final surgical defect; B: illustrates the final defect with planned incision lines and the orientation of the glabellar musculature; C: shows the repair immediately after surgery; D: taken six months post-operatively, demonstrates minimal scarring that blends seamlessly with the natural glabellar rhytides.

A 66-year-old female active smoker with a history of squamous and basal cell carcinomas underwent Mohs micrographic surgery for a nodular basal cell carcinoma at the junction of the right superior nasal sidewall and medial canthus. The preoperative lesion measured 1.3 x 1.2 cm. Following the first stage of Mohs excision, residual tumor was detected on microscopic examination, necessitating a second stage. Frozen section analysis after stage two confirmed complete excision, leaving a final surgical defect of 1.5 x 1.6 cm (Figure 1).

Reconstruction planning considered the defect’s size, involvement of multiple cosmetic subunits, and proximity to the medial canthus. Direct approximation was unsuitable due to the defect’s size and potential tension on the medial canthus. Secondary intention healing was deemed suboptimal due to the patient’s smoking history and associated wound healing risks. A full-thickness skin graft was also less desirable because of the smoking history and its inferior tissue match compared to a local flap. Consequently, a myocutaneous V-to-Y advancement flap was chosen.

Incision were designed to mimic the natural glabellar frown lines. The medial aspect was incised perpendicular to the surface and carried through the glabellar musculature to the periosteum. The lateral side of the flap was incised similarly, with subcutaneous fat undermined above the procerus muscle, serving as the vascular pedicle. The tissue was undermined off the periosteum along the medial incision line to maximize flap mobility.

The flap was advanced to close the primary defect and secured with a 4-0 absorbable suture through both muscle and dermis at the flap edge and defect margin. Additional subcutaneous and dermal 4-0 sutures were placed, and the epidermis was closed with 5-0 fast-absorbing sutures (Figure 1).

Postoperative care included cleaning the area, applying ointment, and dressing with a dry sterile bandage. The patient was instructed to maintain the dressing for 24 hours, followed by daily cleansing, ointment application, and dressing changes. At the 10-day follow-up, the area exhibited good healing with well-approximated edges. At six months, the scar was minimally visible and aligned with the natural glabellar rhytides (Figure 1).

## Discussion

The island pedicle is a type of advancement flap that, unlike other advancement flaps, is not undermined beneath the flap body. Instead, the skin remains attached to its underlying blood supply via a subcutaneous stalk and is advanced over the surgical defect. These flaps are particularly useful in areas where tissue conservation is critical or blood supply is sparse [9]. However, a limitation of this flap is its reliance on regions with sufficient subcutaneous tissue. Consequently, variations of this flap utilizing underlying musculature, such as the nasalis sling flap, have been developed [8].

The nasalis sling flap is a myocutaneous V-Y advancement flap that leverages an underlying muscle’s blood supply to repair defects in relatively avascular areas, such as the distal nose. This flap is supplied by the lateral nasal branch of the angular artery [10]. By undermining submuscularly on the medial aspect while maintaining a muscular pedicle attachment laterally, the flap retains mobility while preserving its vascular supply [11]. This adaptation allows the use of island pedicle flaps in regions with minimal subcutaneous tissue. In our case, a myocutaneous flap was an excellent choice, as the repair involved an area with relatively sparse subcutaneous fat in a patient who was an active smoker.

The nasal bridge has less subcutaneous tissue compared to areas like the cheek or lip, where island pedicle flaps are typically performed. However, the underlying glabellar musculature, including the procerus, corrugator supercilii, and frontalis muscle, is richly vascularized superiorly by the supratrochlear artery [12]. We hypothesized that this vascular source could be utilized similarly to the nasalis sling flap. By incising through the medial aspect of the glabellar complex down to the periosteum and leaving the superolateral muscular stalk intact, this tissue reservoir can be used to reconstruct a defect while preserving a vascular pedicle to enhance tissue perfusion. Given our patient’s active smoking status, utilizing the glabellar musculature rather than a conventional advancement flap was theorized to improve the flap’s survival rate, as suggested by previous studies [13]. Additionally, incorporating the underlying muscle likely enhanced survival, while the overlying glabellar skin provided an excellent tissue texture and color match, with a comparable sun exposure history to the defect site.

Our case presented several challenges: involvement of multiple cosmetic subunits, a free margin, and the patient’s active smoking status. Second-intention healing was not a viable option due to the heightened risk of poor wound healing associated with smoking [1,5]. Webbing of the medial canthus was another potential complication. Moreover, a full-thickness skin graft was deemed suboptimal, given the combination of active smoking history and poor tissue color and texture match. Shaping the flap in alignment with the natural glabellar rhytides was preferred over other local flaps, such as transposition flaps, which can result in unnatural, geometric scar lines. By incorporating muscle into the flap, we anticipated improved survivability, especially in a smoker. Based on our case, we hypothesize that these unique aspects of our flap contributed to the patient’s excellent cosmetic outcome. This novel flap may serve as a reliable and safe option for surgeons facing similar surgical dilemmas.

## Conclusions

The island pedicle flap is an effective option for tissue conservation in areas with limited blood supply. Traditional V-Y advancement flaps rely on sufficient subcutaneous tissue, restricting their use in regions like the nasal bridge. The nasalis sling flap addresses this limitation by incorporating myocutaneous elements to repair defects of the distal nose. Our approach utilizes the vascular-rich glabellar musculature, supplied by the supratrochlear artery, to facilitate repairs on the nasal bridge, particularly in patients at higher risk for poor wound healing. Moreover, unlike traditional flaps used in this area, such as the rhombic transposition flap, which can result in unnatural geometric scar lines, our flap produces a scar that blends seamlessly with the natural glabellar rhytides. It also offers a superior tissue texture and color match compared to a full-thickness skin graft. We hypothesize that the incorporation of myocutaneous elements improves tissue survival compared to alternative techniques. Based on our case, this method provides a robust blood supply, minimal tension, and excellent cosmetic outcomes, making it a valuable repair option for patients with similar defects and risk factors.
